# Dyslipidemia, Cholangitis and Fatty Liver Disease: The Close Underexplored Relationship: A Narrative Review

**DOI:** 10.3390/jcm13092714

**Published:** 2024-05-05

**Authors:** Salvatore Greco, Michele Campigotto, Andrea D’Amuri, Nicolò Fabbri, Angelina Passaro

**Affiliations:** 1 Department of Translational Medicine, University of Ferrara, Via Luigi Borsari 46, 44121 Ferrara, FE, Italy; grcsvt@unife.it; 2 Department of Internal Medicine, Ospedale del Delta, Via Valle Oppio 2, 44023 Lagosanto, FE, Italy; 3 Gastroenterology and Digestive Endoscopy Unit, ASUGI, Cattinara University Hospital, Strada di Fiume 447, 34149 Trieste, TS, Italy; campigottomichele@gmail.com; 4 General Medicine Unit, Medical Department, ASST Mantova, Ospedale Carlo Poma, Strada Lago Paiolo 10, 46100 Mantova, MN, Italy; dmrndr@unife.it; 5 Department of General Surgery, Ospedale del Delta, Via Valle Oppio 2, 44023 Lagosanto, FE, Italy; n.fabbri@ausl.fe.it

**Keywords:** dyslipidemia, acute cholangitis, primary biliary cholangitis, fatty liver disease, hepatic steatosis, cardiovascular risk

## Abstract

In assessing individual cardiovascular risk, dyslipidemia is known for emerging as a pivotal factor significantly contributing to major cardiovascular events. However, dyslipidemic patients frequently present with concurrent medical conditions, each with varying frequencies of occurrence; cholangitis, whether acute or chronic, and hepatic steatosis, along with associated conditions, are strongly associated with specific forms of dyslipidemia, and these associations are reasonably well elucidated. Conversely, evidence linking biliary disease to hepatic steatosis is comparatively scant. This narrative review aims to bridge this gap in knowledge concerning the interplay between dyslipidemia, cholangitis, and hepatic steatosis. By addressing this gap, clinicians can better identify patients at heightened risk of future major cardiovascular events, facilitating more targeted interventions and management strategies. The review delves into the intricate relationships between dyslipidemia and these hepatic and biliary clinical conditions, shedding light on potential mechanisms underlying their associations. Understanding these complex interactions is crucial for optimizing cardiovascular risk assessment as well and devising tailored treatment approaches for patients with dyslipidemia and associated hepatic disorders. Moreover, elucidating these connections empowers clinicians with the knowledge needed to navigate the multifaceted landscape of cardiovascular risk assessment and management effectively. By exploring the intricate relationships between dyslipidemia, cholangitis, and hepatic steatosis (without forgetting the possible clinical consequences of hepatic steatosis itself), this review not only contributes to the existing body of knowledge but also offers insights into potential avenues for further research and clinical practice. Thus, it serves as a valuable resource for healthcare professionals striving to enhance patient care and outcomes in the context of cardiovascular disease and associated hepatic conditions.

## 1. Introduction

The relationship between certain liver and/or biliary tract diseases and dyslipidemia is particularly intricate, and in many aspects it is certainly underexplored. In the current literature, most papers on this topic are observational and show an association between these conditions with an unproven causal relationship. On one hand, it can be stated without fear of contradiction that dyslipidemia is extremely common among individuals with chronic biliary diseases, such as primary biliary cholangitis (PBC). In fact, these patients often present with hypercholesterolemia (ranging from 75% to 95% of cases), without high triglyceride levels, which are frequently within the normal range [1].

Likewise, there is a well-known association between non-alcoholic fatty liver disease (NAFLD, later renamed MASLD, metabolic dysfunction-associated steatotic liver disease), one of the most common chronic liver diseases, and various metabolic disorders, including obesity, type 2 diabetes (T2DM), metabolic syndrome (MS), and dyslipidemia, especially in the developed world [2]. In the case of MASLD, dyslipidemia manifests with increased triglyceride levels and low-density lipoprotein cholesterol (LDL-c) in the serum, as well as reduced high-density lipoprotein cholesterol (HDL-c), placing individuals with MASLD at increased risk of major cardiovascular (CV) events [3].

A comparable situation exists for alcohol-associated liver disease (ALD), which is closely related to metabolic conditions such as T2DM and dyslipidemia and is primarily linked to alcohol consumption. There are no doubts about the etiopathogenesis of this metabolic problem, but it is equally important to implement preventive strategies to reduce the high CV risk associated with these conditions [4]; finally, a new category of MASLD has been added to the new classification, for subjects with contextual alcohol consumption, and has been named MetADL [5].

The relationship between inflammatory biliary tract diseases, both acute and chronic, and MASLD is even more complex, and some cohort studies, especially retrospective ones, have been dedicated to this aspect. For instance, a group of Canadian researchers have conducted a study on 194 subjects (a 1:2 match between subjects with MASLD/PBC and those with only MASLD), concluding that PBC does not appear to worsen the course of MASLD [6]. Meanwhile, some Israeli authors have recently published a multicenter study involving a total of 811 subjects, demonstrating a significant association between the diagnosis of MASLD and acute cholangitis secondary to common bile duct (CBD) gallstones [7].

In 2021, a group of researchers from China retrospectively analyzed a group of 479 patients with PBC, chronic hepatitis B and C, and NAFLD, also including healthy subjects in the analyses, and found that PBC had the lowest rates of hepatic steatosis with higher levels of HDL cholesterol [8]. They suggested that, even if PBC subjects were overall hyperlipidemic, they did not present an increased incidence of atherosclerosis and/or CV events, further adding to the doubts as to the need for lipid-lowering treatments and CV risk assessments of these patients.

The objective of this study will be to partially fill the existing knowledge gap regarding the association between inflammatory biliary tract diseases and dyslipidemia by conducting a literature review on the described topics. It will describe various associations between MASLD, cholangitis (acute or chronic), and dyslipidemia, potentially uncovering relationships between these medical conditions. 

This review potentially marks the first attempt to consolidate these three illnesses and synthesize their pathogenesis; its aim is to aid clinicians in identifying the theoretical predisposing factors associated with heightened CV risk in individuals with these diseases, while also preventing an undue overestimation of such risk.

## 2. Materials and Methods

As stated above, the aim of this review is to analyze the relationships existing between MASLD, cholangitis (both acute and chronic), and dyslipidemia. To achieve our purposes, we searched for all relevant articles from the PubMed and Scopus databases, without time restrictions, according to the following queries: “cholangitis”, “MASLD”, “fatty liver disease”, “hyperlipidemia”, “hyperlipidaemia”, “dyslipidemia”, and “dyslipidaemia”.

Due to the limited sample size of studies involving all three clinical conditions simultaneously (dyslipidemia, hepatic steatosis, and biliary diseases, whether acute or chronic), we considered the main articles regarding associations between two pathologies at a time. For this exact reason, our choice was to conduct a narrative review of the literature; because of the fact that this is not a systematic review and few articles were found, evaluations of methodological quality were not used to exclude papers from the study. 

The articles were initially screened by title from two independent authors (SG and MC) and then by abstract content; they were finally included in the review after revisions by a third author (NF), who resolved eventual conflicts and decided on acceptance/rejection. 

In total, 70 articles were eligible for inclusion in this review, while only 24 of them had the full text available for free. The risk of bias was assessed independently by two reviewers (SG and MC), and eventual conflicts were resolved via discussion. 

No automation tools or machine learning techniques were used in this research.

## 3. Fatty Liver Disease and Dyslipidemia

The two major types of fatty liver disease are represented by ALD and MASLD, which are extremely similar from a histological perspective. They both encompass liver steatosis (whether induced by alcohol or not), steatohepatitis (alcohol-induced, known as ASH, or not, referred to as NASH), progressive fibrosis, cirrhosis, and hepatocellular carcinoma [9,10] and are strongly associated with an increased CV risk [11]. Moreover, MASLD can be considered the hepatic manifestation of the metabolic syndrome, and is typically associated with T2DM, insulin resistance, hypertension, obesity, and dyslipidemia. Indeed, MASLD also shares a pathogenesis with all the aforementioned conditions, predisposing individuals to a higher risk of major adverse CV events (MACEs) [12]. For these reasons, a change in terminology from MASLD to metabolic associated fatty liver disease (MAFLD) was recently proposed, underscoring the active role of metabolic dysfunction over the absence of alcohol use in the pathogenesis of liver disease [13].

Some other studies have examined the role of alcoholic steatohepatitis in determining higher CV risk, concluding that alcohol consumption can effectively lead to a higher incidence of MACEs, as well as pure liver disease [14,15]. However, the association of both types of fatty liver disease with major CV risk factors is profoundly unexplored and further dedicated studies would be needed to investigate these aspects further.

Moreover, the association with each individual CV risk factor appears to differ between ALD and MASLD; some evidence suggests that ALD is more associated with hypertension than MASLD, while other evidence suggests that MASLD is more associated with dyslipidemia [16]. A more recent nationwide survey has further confirmed this strong association between MASLD and dyslipidemia and has suggested a strong association of the former with T2DM than with ALD [17].

As for MASLD and its association with traditional CV disease risk factors, it should be noted that it also presents a significant link with hyperuricemia [18], hypoadiponectinemia [19], hypovitaminosis D [20], and chronic kidney disease (CKD) [21], which come with varying degrees of higher risk for several different illnesses. Moreover, in subjects with MASLD, there is an overproduction of proinflammatory cytokines such as interleukin 6 (IL-6) or tumor necrosis factor alpha (TNF-α) and procoagulant factors (fibrinogen; plasminogen activator inhibitor-1), or adhesion molecules (vascular adhesion protein-1), predisposing individuals to chronic low-grade inflammation [22,23].

The development of fatty liver disease and its progression are associated with the activity of several proteins secreted by the liver, called hepatokines, which contribute to an altered accumulation of fat in the liver itself. Theoretically, a fatty liver works differently from a healthy one and is subject to dysregulation in terms of the secretion of a series of proteins (such as fetuin-A and B, ANGPTL3, FGF21, selenoprotein P, and follistatin, which are increased in fatty livers, while SHBG levels are decreased compared with those in healthy livers) [24].

Similarly, some other proteins secreted by the adipose tissue are intricately involved in liver dysregulation, contributing to disease development and progression; among these, we can find adiponectin (decreased in subjects with fatty liver diseases and inversely correlated with the severity of hepatic steatosis), ghrelin (its lower levels in MASLD subjects are associated with insulin resistance), leptin (which is positively correlated with steatosis severity), and resistin, irisin, and visfatin (which are all generally increased in steatotic livers) [25].

The link between MASLD and dyslipidemia is even closer. Recently, it was postulated that multiple cellular mechanisms work simultaneously, causing chronic hepatic inflammation and the progression of liver disease to NASH [26]. Subjects with MASLD experience abnormal lipid metabolism, as well as mitochondrial oxidative injury, endoplasmic reticulum (ER) stress, and altered immune responses [27,28]. This results in an abnormal hepatic storage of triglycerides and cholesterol. 

While triglycerides serve as a form of secure storage and can be used as substrates for energy production and metabolic pathways [29], cholesterol faces a dual fate: on one hand, it is esterified, forming cholesterol esters (CEs), which are typically inert, while on the other hand, it exists in the unesterified form (hepatic free cholesterol), associated with high cellular toxicity, hepatic inflammation, and progressive organ disease [30].

Particularly important is hepatic cholesterol homeostasis, which is regulated by several nuclear transcription factors; among these, three have been associated with MASLD pathogenesis: SREBP-2 (sterol regulatory element binding protein-2), FXR (farnesoid X receptor), and LXR (liver X receptor).

SREBP-2 belongs to the SREBP family, involved in cholesterol and fatty acid synthesis and uptake [31]. It is present in the ER, and, after translocating into the Golgi complex and then into the nucleus, it activates the transcription of HMGCoAR (3-hydroxy-3-methylglutaryl coenzyme A reductase) and LDLR (low-density lipoprotein receptor) [32].

FXR, also known as BAR (bile acid receptor), is a nuclear receptor strictly involved in bile acid, lipid, and glucose homeostasis [33]; it up-regulates SR-B1 (scavenger receptor class B type 1), increasing the uptake of circulating HDL (high-density lipoprotein), prevents cholesterol conversion into bile acids by inhibiting a specific hepatic cytochrome (CYP7A1), and promotes the removal of triglycerides by increasing β-oxidation and decreasing lipogenesis [34].

The last transcription factor is represented by LXRs, which are important in the regulation of cholesterol, fatty acid, and glucose homeostasis and include the two isoforms: LXRα and LXRβ. Oxysterols, the oxygenated derivatives of cholesterol, naturally bind to LXRs [35], and, after activation, LXRα forms a heterodimer with RXR (retinoid X receptor), resulting in reverse cholesterol transport and increased HDL levels, but also cholesterol excretion and LDLR degradation [36,37].

Regarding cholesterol esterification, the key player is ACAT2 (acyl-CoA:cholesterol acyltransferase enzyme 2), a transmembrane protein of the ER of the liver [38]; it integrates newly formed cholesterol esters into the ER membrane, which are then incorporated into apolipoprotein B (ApoB) or extruded, forming lipid droplets, the universal storage organelles found in most cells, facilitating lipid uptake, distribution, and storage/use coordination [39]. When free cholesterol is required, ACAT2 is downregulated by a specific hydrolase (nCEH, neutral cholesterol ester hydrolase) that hydrolyzes cholesterol esters to free cholesterol [40].

In subjects with MASLD, there is often an accumulation of liver fat, resulting from a misbalance in several metabolic pathways, such as the uptake of circulating lipids, hepatic de novo lipogenesis, fatty acid oxidation, and lipid export [41]. Alterations in the hepatic uptake of triglycerides and de novo lipogenesis lead to increased triglyceride synthesis and an elevated secretion of very-low-density lipoproteins (VLDLs), which are known to contribute to atherogenesis and increase CV risk [42].

Insulin resistance, typically associated with MASLD, further promotes lipoprotein abnormalities, causing hyperglycemia, ectopic lipid accumulation, endothelial dysfunction, and dyslipidemia as well [43]. 

Notably, a significant association between MASLD and monogenic dyslipidemia warrants attention. Specifically, individuals with heterozygous familial hypercholesterolemia (HeFH), the most prevalent monogenic dyslipidemia, exhibit a MASLD prevalence comparable to that of the general population [44]. On the other hand, a distinctive monogenic disorder, lysosomal acid lipase (LIPA) deficiency, emerges as a rare but impactful contributor to severe steatosis progressing to juvenile metabolic cirrhosis [45].

LIPA, a key enzyme in the LDL receptor pathway in hepatocytes after its internalization via endocytosis, plays a crucial role. Essentially, it facilitates the separation of the LDL receptor from the bound lipoprotein within the lysosome; this enables the subsequent expression of the LDL receptor on the hepatocyte surface. In cases of LIPA deficiency, the normal progression of the LDL receptor–lipoproteins complex through this pathway is disrupted. As a result, lipoproteins accumulate at the lysosomal level, precipitating the genesis of steatotic disease at an earlier stage, proportional to the degree of impairment of LIPA function. Furthermore, owing to the diminished expression of LDL receptors on the hepatocyte surface, individuals with LIPA deficit also manifest a hypercholesterolemic phenotype comparable to that observed in familial hypercholesterolemia (HeFH) [45].

## 4. Cholangitis and Dyslipidemia

Regarding the association between biliary pathology (both acute and chronic) and dyslipidemia, there are currently few studies in the literature that establish a strong connection. As mentioned earlier, dyslipidemia is common in individuals with chronic biliary pathologies such as PBC, showing a prevalence of hypercholesterolemia over hypertriglyceridemia. LDL-c levels are generally higher than desirable, theoretically indicating a higher risk of CV events [1]. However, these individuals also exhibit elevated HDL-c levels, mainly due to increased circulating levels of ApoA-1 (apolipoprotein A1), the primary component of HDL lipoproteins [46]; this condition paradoxically associates with a reduced incidence of CV events, regardless of elevated LDL-c levels.

The reason for this must be sought through other factors; individuals diagnosed with PBC also exhibit increased circulating levels of adiponectin (in contrast to patients with MASLD) [47] and lipoprotein-X (Lp-X), which some studies have suggested to be implicated in reducing CV risk and atherosclerosis [46,48].

Adiponectin, discovered in the mid-1990s, is one of the main cytokines produced by adipose tissue and is effectively classified as a hormone due to its involvement in the regulation of cell survival, growth, and apoptosis [49]. Among the multiple effects of adiponectin, it regulates PPAR-γ (peroxisome proliferator-activated receptor gamma), a second-type nuclear receptor implicated in lipid and glucose metabolism, adipogenesis, and inflammation. Excess adipose tissue is particularly associated with reduced adiponectin production, leading to increased hepatic glucose production and uptake, elevated triglycerides, simultaneous reductions in HDL and VLDL catabolism, and an increase in various inflammation markers [50]. Notably, an elevation in adiponectin levels through the administration of PPAR-γ agonists (i.e., thiazolidinedions), but also with natural garlic extracts, Zataria multiflora, cobalt, or L-cysteine, has shown to be a promising approach in lowering obesity-related disease in both humans and mice, and their role deserves to also be evaluated more precisely in cholestatic diseases-associated CV risk [51].

Another key aspect in the complex understanding of CV risk associated with chronic biliary disease is represented by Lp-X. This lipoprotein is rich in phospholipids, bile acids, albumin, and unesterified cholesterol at a density similar to that of LDL. Although the two are structurally similar, Lp-X presents apolipoproteins such as ApoA-1 and Apo-E but lacks Apo-B, which is present in LDL, where it acts like a ligand for its main receptors; this prevents such interactions and inhibits Lp-X clearance by hepatic receptors [52].

In a functionally intact liver, the enzyme LCAT (lecithin-cholesterol acyltransferase) converts free cholesterol in Lp-X into esterified cholesterol [53], while in individuals with PBC, the activity of this enzyme is significantly reduced due to cholestasis induced by biliary pathology, leading to a substantial increase in Lp-X levels [54].

Elevated levels of this lipoprotein not only increase in individuals with primary biliary disease but also in cases of acute cholestasis such as cholangitis, with mechanisms not yet fully understood. It has been hypothesized that, following the cholestatic phase, part of the bile is drawn into the plasma compartment, where, in contact with albumin, lipoproteins rearrange to form Lp-X, presenting as vesicles [55]. In practice, the structural similarities between LDL and Lp-X can configure a picture of hyperlipidemia not supported solely by an increase in LDL-c but also by Lp-X. However, it is essential to note that this is an extremely rare condition.

In general, at least four conditions have been described in which a high concentration of Lp-X leads to the development of dyslipidemia: (a) cholestasis (regardless of triggering causes), (b) LCAT deficiency, (c) infusion of lipid-rich solutions, and (d) graft vs. host disease in liver transplant patients [56].

Treatment of high lipid levels due to Lp-X is primarily based on resolving the underlying cholestatic diseases, as this prevents lipid particles from migrating into the blood, and because the formation of Lp-X is stopped [57]. Depending on the levels of LDL-C left, there is a theoretical role for lipid-lowering drugs (i.e., statins), even if the recommendation in this case is as strong as that for the general population. Ezetimibe is not advisable as an alternative, because in case of cholestasis, intestinal cholesterol absorption does not contribute to high serum lipid levels because of insufficient micellar formation [46]. 

In contrast to fatty liver disease, where Lp-X is formed in circulation, in cholestatic liver diseases, it originates from the liver. Therefore, a reduction in or limited availability of chylomicrons with a fat-restricted diet along with angiotensin-II receptor blocker administration [58,59], or the administration of a combination of nicotinic acid and fenofibrate [60], which represent a promising approach, need to be re-evaluated in this setting.

In recent years, instead, several LCAT-raising biologic approaches (recombinant human LCAT or rhLCAT), direct or indirect gene therapies, peptide activators, and small molecules are currently under development as potential therapeutics for cholestasis, especially when it reaches such high levels that it causes hyperviscosity syndrome, requiring plasmapheresis [61].

## 5. Fatty Liver Disease and Cholangitis

### 5.1. MASLD and PBC

The relationship between MASLD and biliary diseases, both primary and acquired, is undoubtedly more complex, and, in terms of several aspects, underexplored. While the pathogenesis of MASLD is increasingly clear, many uncertainties exist regarding that of primary biliary cholangitis (PBC). Even if immune-mediated damage seems to be partly involved in the genesis of PBC, recent evidence has suggested a possible role of viral pathogens and bile acids, which would be toxic if excessively retained [62].

On the other hand, it is known that MASLD can potentially progress over the years, and behind this potential progression, factors such as endotoxins of intestinal origin capable of activating hepatic innate immunity, inducing the proinflammatory state typical of steatosis pathology, are theoretically involved [63].

PBC is a condition that classically predisposes individuals to chronic biliary stasis, resulting in an inflammatory state affecting small- and medium-sized bile ducts, with potential progression to cirrhosis [64]. One of the most typical markers of damage is represented by alkaline phosphatase (ALP), a protein capable of phosphorylating certain compounds in an alkaline environment, including endotoxins [65]. From this assumption, therefore, a theory has emerged that cholestatic disease with a consequent increase in serum ALP could theoretically protect against a chronic condition that is affected by the accumulation of endotoxins, such as MASLD. The only study dedicated to the pathogenetic correlation between MASLD and PBC is the one by Iluz-Freundlich and colleagues mentioned earlier, which did not establish any causal link between the two examined conditions [6]; some commonalities in gut microbiota alterations in MASLD and PBC are described in the current literature, with changes in the *Bacteroidetes* to *Firmicutes* ratio, but this evidence is also found in many other hepatic conditions [66] and, therefore, it is not possible to express a definite opinion on how MASLD and PBC interact and influence each other [67]. A better understanding of this interaction could be developed based on studies describing treatment efficacy.

Based on our current knowledge about therapies in hepatobiliary diseases, farnesoid X receptor (FXR) agonists (i.e., Obethicolic acid- OCA), which act through an increase in the transcription of various genes, including intestinal FGF-19, have shown benefits in both PBC, modulating the production and secretion of bile acids, and NASH individuals, potentially mitigating lipid dysregulation, influencing extracellular matrix reorganization and suppressing hepatic stellate cell activation. This potential efficacy and the promising use of OCA in both diseases [68] could lead to a speculation of the FXR–FGF-19 axis as a linker between these two diseases, with a potentially safe and effective treatment. 

Moreover, ursodeoxycholic acid (UDCA), the first-line therapy for PBC, has also shown surprising therapeutic potential in NAFLD with several mechanisms, such as improvement in cellular autophagy, apoptosis, and mitochondrial functions. These actions are based on its direct or indirect effect, targeting the farnesoid X receptor (FXR), further supporting the speculation of its role in both diseases. However, further evidence from animal models and human studies on PBC/MAFLD patients is required.

### 5.2. MASLD and Acute Biliary Disease

As for the relationship between MASLD and acute biliary disease, it is necessary to take a step back and discuss the causes that can lead to inflammation of the biliary ducts, which is represented by cholangitis. In most cases, biliary inflammation is due to obstructive processes of both a benign and malignant nature, complicated by concurrent bacterial, or, rarely, parasitic infection [69]. Gallstone disease, mainly of a gallbladder origin, is the most frequent cause of acute cholangitis, as well as other gastrointestinal diseases with high morbidity and mortality, such as acute cholecystitis and acute pancreatitis. Treatments therefore consist of traditional biliary stone extraction through endoscopic retrograde cholangiopancreatography (ERCP) and antibiotic therapies in the majority of cases.

In recent years, MASLD has been the subject of various association studies, which have demonstrated its association with significantly different conditions, such as obesity, insulin resistance, and T2DM, as explained in the introductory part of the study, as well as with community-acquired pneumonia [70], colonic polyposis [71], and breast cancer [72]. Recently, MASLD has also been associated with an increased severity of acute pancreatitis [73] and acute cholangitis [7], both conditions that are certainly more homogeneous and conceptually more related to liver disease; in the latter case, in particular, MASLD has shown a significant association with acute biliary disease, especially in individuals presenting to the physician with symptomatic gallstones.

An even more recent population-based study by US researchers [74] has demonstrated a strong association between acute liver and biliary conditions, resulting in a significant increase in hospitalization lengths of stay, as well as increased in-hospital mortality. 

There are many commonalities between MASLD and acute cholangitis: the latter is also associated with T2DM and insulin resistance [75], obesity (which is, in turn, strongly predisposes individuals to the formation of gallstones) [76], and intestinal dysbiosis (understood as an imbalance of intestinal bacterial flora) [77]. The most likely hypothesis is that primary bacteremia of the presumed gastrointestinal origin in MAFLD patients, supporting the role of the gut–liver axis [78], can be associated with the low-grade inflammation generated chronically in the liver condition (MASLD) and may somehow predispose individuals to inflammation in the small- and medium-sized bile ducts. Targeting gut microbiota for preventing acute infectious processes is thus a very compelling hypothesis; however, no evidence for the use of probiotics or antibiotics as a form of prevention of acute cholangitis is described to date in MASLD patients.

Another hypothetical genetic mechanism by which obesity or NAFLD leads to more aggressive inflammation was described in experimental rats, where an intense ductular inflammation was shown in response to a high-fat diet in liver-specific E-cadherin knockout [79]. Whether or not this interesting mechanism could be a target of potential treatment is still to be investigated, but it clearly describes that both genetic and environmental factors could interact with each other in promoting inflammation. No link between cholangitis and well-known genes involved in MASLD promotion (i.e., PNPLA3, TM6SF2, or MBOAT7) has been described to date.

Other associated factors can link MAFLD and cholangitis, such as metabolic diseases in general, which may contribute to the development of the acute event. This, in fact, would worsen the abdominal picture, resulting in a higher rate of hospitalization, morbidity, and mortality in affected individuals, as described earlier.

## 6. Discussion

The relationships between conditions involving the biliary system, hepatic steatosis (whether alcohol-induced or not), and dyslipidemia are much more intricate than commonly thought. The global incidence of conditions such as overweight and obesity, diabetes, hypertension, metabolic syndrome, and dyslipidemia contributes to the increasing burden of metabolic diseases, annually raising mortality from CV causes, of which these pathologies are the main risk factors.

Our article aims primarily to raise awareness within the scientific community on considering the association between these seemingly heterogeneous pathologies, which, in reality, seem to form a unique entity often requiring a holistic approach to improve overall treatment.

As detailed in the preceding chapters, both MASLD and cholangitis (in acute or chronic forms such as PBC) are more or less evidently associated with dyslipidemia, which itself poses a high CV risk and often requires appropriate treatment to be reduced [69]. 

Knowledge regarding the association between MASLD (and its evolving condition, NASH) and dyslipidemia is well established in the literature; recently, some Greek researchers delved into the role of treatment with lipid-lowering drugs (specifically statins) in populations affected by both MASLD and NASH, concluding on the safety and usefulness of statin pharmacological treatment. Statins were found to reduce both the overall CV risk in these individuals [70] and the theoretical possibility of progression to more aggressive forms of liver disease, such as hepatocellular carcinoma (HCC) [71].

On the other hand, when it comes to conditions affecting the biliary tree, the discourse is undoubtedly more complex and requires clarification. Chronic conditions such as PBC are associated with substantial hormonal changes, such as hyperadiponectinemia, leading to elevated levels of serum HDL and an increased catabolism of VLDL, resulting in normalized triglyceride levels [47,48]. However, elevated cholesterol levels and increased LDL-c are common in these individuals, indicating an overall higher CV risk [1]. 

This complex scenario suggests that individuals with PBC theoretically appear to be less predisposed to developing major CV events despite the aforementioned hypercholesterolemia. Adding to the complexity is the role of obeticholic acid (OCA), an FXR ligand recently approved for PBC treatment, which is associated with a further increase in LDL-c, especially in subjects with concurrent NASH [72]; there are controversial data on this matter come from trials dedicated to individuals exclusively affected by PBC [73,74]. Ursodeoxycholic acid (UDCA), considered the main treatment for PBC, has shown an improving pattern regarding hypercholesterolemia, reducing LDL-c and VLDL-c, although with neutral effects on HDL-c or triglycerides [75].

According to the current literature, there is not enough evidence regarding an increased cardiovascular risk in patients with PBC only; as early as in 2018, Suraweera et al., in their systematic review [76], stated that, in subjects with PBC and without other concomitant features of metabolic syndrome, individual risk/benefit discussion on lipid-lowering treatment should necessarily be considered.

Among the other illnesses potentially heightening the overall cardiovascular risk factors in PBC subjects, we can find hypertension and T2DM (besides dyslipidemia, as already indicated in the paragraph above), with hypertension playing an apparently significant role in worsening these patients’ outcomes; in particular, anti-sp100 antibodies considered specific for PBC were found associated with adverse outcomes in PBC subjects [77].

Moreover, recent evidence suggests that the gut microbiota is also able to play a crucial role in the pathogenesis of chronic biliary conditions such as PBC itself, contributing to a dysregulation of bile acid metabolism and the immune response, and to chronic inflammation [78]; considering that the gut microbiota was also associated with increased cardiovascular risk [79], its potential role in poor outcomes in PBC subjects should not be ignored.

When discussing dyslipidemia associated with acute cholestatic events (due to concurrent inflammation of the biliary pathways), it is crucial to consider the elevation of Lp-X, instead. This lipoprotein, due to its structural similarity to LDL, can mimic a condition of false hyperlipidemia, making it extremely challenging to distinguish it from actual LDL-c hyperlipidemia; this can lead to a sort of overtreatment of dyslipidemia associated with cholestasis and, theoretically, a risk of drug-induced toxicity due to reduced biliary excretion [54].

Substantially, in the case of dyslipidemia associated with biliary pathology, several factors must be considered. Firstly, the nature of the condition leading to the increased lipid molecules must be assessed. For individuals with PBC, it is necessary to evaluate both the most suitable way to reduce the CV risk associated with concurrent dyslipidemia and that related to the biliary pathology that predisposes individuals less to a worsening of the lipid profile. On the other hand, individuals with cholestasis from acute cholangitis must undergo further evaluation, taking into account the possibility of a concurrent increase in Lp-X, which is not necessarily associated with an increased CV risk.

The elucidation of intricate relationships between liver and biliary tract diseases and dyslipidemia presented in this manuscript carries profound clinical implications across various domains of patient care and healthcare policy. By synthesizing the intricate associations between metabolic dysfunction-associated steatotic liver disease (MASLD), cholangitis, and dyslipidemia, this review sets the stage for a deeper understanding of these complex medical conditions and their interplay. Below, we outline the clinical implications derived from our analysis.

Treatment Guidance. The comprehensive understanding of the associations between MASLD, cholangitis, and dyslipidemia offers valuable insights into tailoring treatment strategies for affected individuals. Clinicians can leverage this knowledge to optimize therapeutic interventions, such as lipid-lowering medications, in patients with concomitant liver and biliary tract diseases. Moreover, the identification of specific risk factors and underlying pathophysiological mechanisms informs personalized treatment approaches, thereby enhancing treatment efficacy and patient outcomes.

Diagnostic Criteria. The delineation of associations between the aforementioned conditions underscores the importance of integrated diagnostic assessments in clinical practice. By recognizing the intricate links between these conditions, clinicians can refine diagnostic criteria, leading to earlier detection and intervention. Furthermore, the identification of novel biomarkers indicative of disease pathology facilitates accurate diagnostic evaluations, minimizing diagnostic uncertainty and enabling timely therapeutic interventions.

Patient Management Strategies. The elucidation of complex relationships between metabolic liver diseases, biliary tract disorders, and dyslipidemia informs holistic patient management strategies aimed at optimizing health outcomes. Clinicians can develop tailored management plans that address both the liver and cardiovascular health needs of affected individuals. Additionally, the integration of multidisciplinary care approaches, including lifestyle modifications, pharmacotherapy, and close monitoring, enhances the overall management of these complex medical conditions.

Healthcare Policy Implications. The insights gleaned from this review have far-reaching implications for healthcare policies and guidelines. By recognizing the intricate associations between the medical conditions described, policymakers can advocate for integrated care models that prioritize early detection, comprehensive diagnostic assessments, and evidence-based management strategies. Furthermore, the integration of preventive measures targeting modifiable risk factors, such as obesity and dyslipidemia, can mitigate disease burden and reduce healthcare expenditures in the long term.

Patient Education and Empowerment. The dissemination of knowledge derived from this review could empower patients to actively engage in their healthcare journey and make informed decisions about their health. By providing educational resources and promoting health literacy, patients gain a deeper understanding of the complex relationships between liver and biliary tract diseases, dyslipidemia, and cardiovascular risk. Moreover, patient-centered educational initiatives foster shared decision-making processes, promoting collaborative partnerships between patients and healthcare providers.

In conclusion, the clinical implications outlined herein underscore the transformative potential of integrating the intricate associations between metabolic liver diseases, cholangitis, and dyslipidemia into clinical practice and healthcare policy. By leveraging this knowledge, clinicians can enhance diagnostic accuracy, optimize treatment strategies, and improve patient outcomes, ultimately advancing the quality of care for individuals affected by these complex medical conditions. Furthermore, starting from the knowledge of the pathophysiological mechanisms underlying these intricate illnesses, they will have a greater likelihood of assessing the cardiovascular risk of patients more accurately.

## 7. Conclusions

Fatty liver disease (and the conditions that may result from it), cholangitis (both acute and chronic), and dyslipidemia, despite being highly heterogeneous conditions, are strongly interrelated. Assessing the cardiovascular risk of individuals with dyslipidemia and one of these conditions is challenging and necessitates understanding the underlying cause. The risk of overtreatment in some conditions associated with hyperlipidemia is real, and clinicians must grapple with the difficult task of distinguishing between individuals at higher and lower risk of developing future major cardiovascular events.

## Data Availability

Not applicable.
